# Treatment Sequencing in Renal Ewing's Sarcoma With Inferior Vena Cava (IVC) Thrombus: A Comparative Case-Based Analysis

**DOI:** 10.7759/cureus.116439

**Published:** 2026-09-17

**Authors:** Shiv Charan Navriya, Aquib Javed M Patel, Jitendra S Rathor, Gautam Choudhary, Sudeep Khera, Aasma Nalwa, Arjun S Sandhu

**Affiliations:** 1 Department of Urology, All India Institute of Medical Sciences, Jodhpur, Jodhpur, IND; 2 Department of Pathology and Laboratory Medicine, All India Institute of Medical Sciences, Jodhpur, Jodhpur, IND

**Keywords:** inferior vena cava tumour thrombus, multimodal intervention, radical nephrectomy with ivc thrombectomy, renal ewing's sarcoma, retroperitoneal sarcoma surgery

## Abstract

Renal Ewing's sarcoma with inferior vena cava (IVC) tumour thrombus is a rare and aggressive malignancy that may mimic renal cell carcinoma (RCC) on imaging, creating diagnostic and therapeutic challenges. We report two young female patients presenting with large renal masses and level II IVC tumour thrombus who were managed using contrasting treatment sequences. In the first patient, image-guided biopsy established the diagnosis, allowing neoadjuvant systemic chemotherapy that resulted in substantial tumour and thrombus downsizing and facilitated subsequent surgical resection. In the second patient, persistent haematuria and progressive anaemia prompted urgent upfront surgery, which was followed by significant postoperative morbidity and delayed initiation of systemic chemotherapy. These contrasting cases highlight the potential value of early tissue diagnosis in selected clinically stable patients with atypical renal masses and emphasise that treatment sequencing should be individualised according to clinical status, tumour extent, thrombus burden, and multidisciplinary assessment.

## Introduction

Primary Ewing's sarcoma of the kidney is an exceptionally rare and aggressive tumour that predominantly affects young adults [1,2]. Although Ewing's sarcoma is one of the more common malignant tumours of bone and soft tissue in children and young adults, primary Ewing's sarcoma arising at an extraosseous site such as the kidney is uncommon. Only a limited number of cases of renal Ewing's sarcoma with inferior vena cava (IVC) tumour thrombus have been reported in the literature. Renal Ewing's sarcoma is characterised by rapid growth and a high propensity for early metastasis, most frequently to the lungs, bones, and lymph nodes [3]. Common clinical manifestations include abdominal or flank pain, haematuria, and a palpable abdominal mass, although these features are nonspecific and may overlap with other renal malignancies.

Radiologically, renal Ewing's sarcoma may mimic renal cell carcinoma (RCC), presenting as a large heterogeneous renal mass with areas of necrosis or haemorrhage [4]. The presence of IVC tumour thrombus is particularly important because it indicates extensive venous involvement and has major implications for operative planning, including the need for vascular control, thrombectomy or venous resection, and, in selected cases, more complex vascular procedures. IVC thrombus also reflects locally advanced disease and may contribute to the overall prognostic assessment. This radiological overlap and the rarity of renal Ewing's sarcoma can result in diagnostic uncertainty. Definitive diagnosis requires histopathological assessment supported by appropriate immunohistochemical and, where available, molecular studies to distinguish Ewing's sarcoma from other small round cell tumours.

Given the aggressive biology and systemic nature of Ewing's sarcoma, treatment generally involves multimodal therapy incorporating systemic chemotherapy and definitive local control with surgical resection. Ewing's sarcoma is considered a chemotherapy-sensitive malignancy, providing a rationale for systemic therapy before definitive surgery in selected patients with locally advanced disease. However, due to the rarity of renal Ewing's sarcoma, particularly in the presence of IVC tumour thrombus, there are no standardised treatment guidelines regarding the optimal sequence of biopsy, chemotherapy, and surgery. In clinically stable patients in whom the diagnosis is uncertain and histological confirmation is likely to alter management, a biopsy-first approach may therefore facilitate early initiation of systemic therapy and multidisciplinary treatment planning.

We present two cases of renal Ewing's sarcoma with IVC tumour thrombus managed using contrasting treatment sequences: biopsy followed by neoadjuvant chemotherapy versus upfront surgery followed by adjuvant chemotherapy. These cases highlight the clinical challenge of decision-making in patients with radiologically presumed RCC and emphasise the potential role of early tissue diagnosis and individualised treatment sequencing in this rare clinical setting.

## Case presentation

Case 1

A woman aged 26 years presented with lower abdominal pain radiating to the left flank and decreased urine output for three days. Table 1 shows the baseline investigations of the first patient showing decreased haemoglobin with marginally higher serum creatinine.

**Table 1:** Baseline investigations of the first patient The first patient had low haemoglobin and serum creatinine at a borderline high level.

Parameter	Obtained value	Reference value
Haemoglobin	8.1 g/dL	11-15 g/dL
Serum creatinine	1.03 mg/dL	0.45-1 mg/dL

Contrast-enhanced imaging of the abdomen and thorax revealed a large left renal mass measuring 18.2 × 7.2 × 5.3 cm with level II IVC thrombus as shown in Figure 1A-1B. The mass demonstrated >180° aortic involvement with loss of fat planes with the duodenum and psoas muscle while maintaining planes with the spleen and jejunum. No distant metastases were detected.

**Figure 1:**
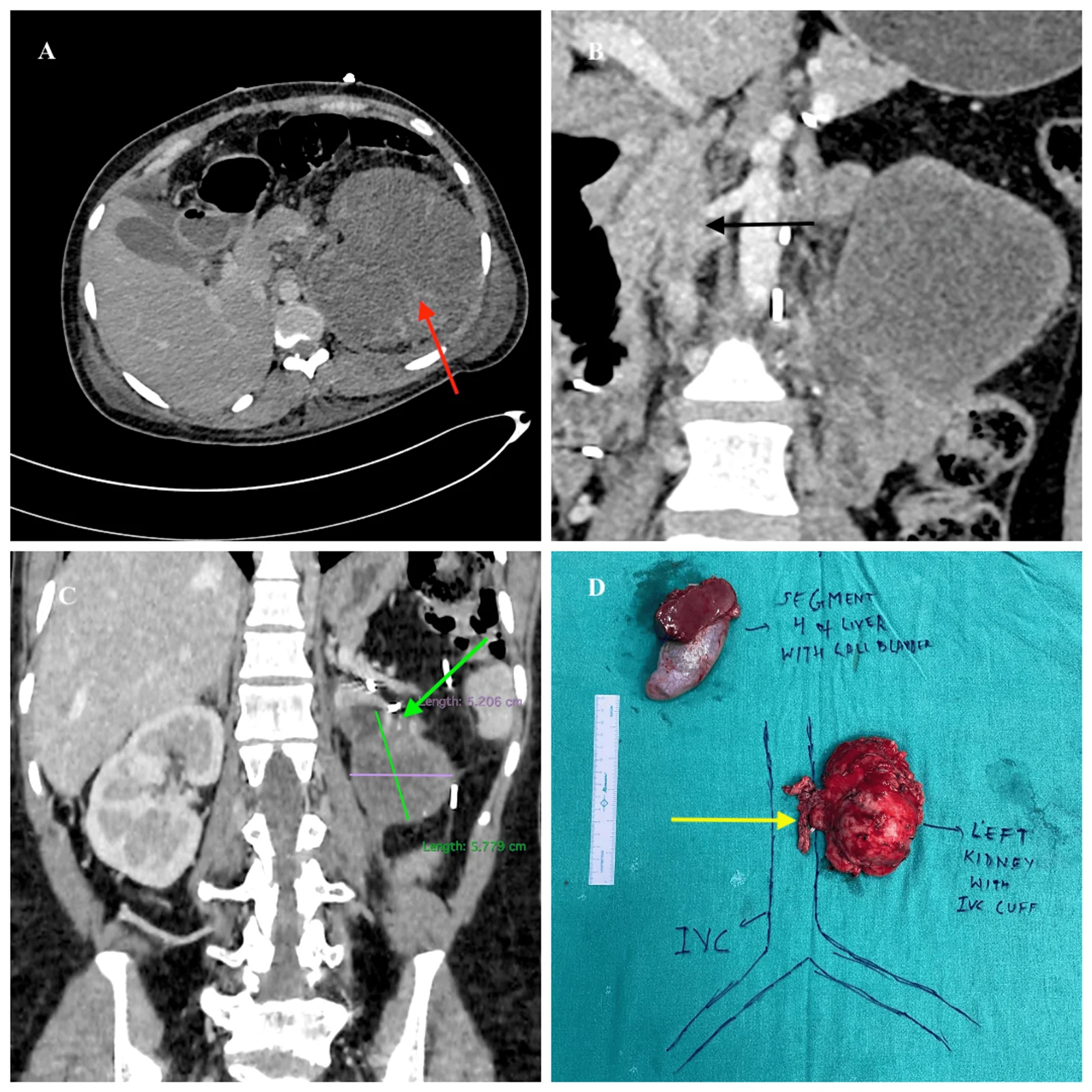
CT scan and postoperative specimen of Case 1 (A) Axial CT scan cut image with the red arrow showing the left renal mass. (B) Coronal CT scan with the black arrow showing the IVC thrombus. (C) Coronal CT scan with the green arrow showing the post-chemotherapy reduction in mass size. (D) Postoperative specimen with the yellow arrow showing the left kidney with the IVC cuff and segment 4 of the liver with the gallbladder. IVC: inferior vena cava

Given the extensive local involvement and suspicion of a non-RCC malignancy, a CT-guided biopsy was performed. On immunohistochemistry (IHC), the tumour cells were positive for CD99 and NKX2.2 and negative for CD45, cytokeratin 7, cytokeratin 20, PAX8, vimentin, AMACR, CD10, cyclin D1, WT-1, synaptophysin, chromogranin, pan cytokeratin, BCOR, TFE3, Oct3/4, FLI1, TLE1, and SALL4. The Ki-67 proliferation index is 15%.

Following multidisciplinary team discussion, the patient was initiated on a VAC/IE chemotherapy regimen. Vincristine (1.4 mg/m²), doxorubicin (75 mg/m²), and cyclophosphamide (1200 mg/m²) were administered on day 1, while ifosfamide and etoposide were administered on days 1-5, with cycles repeated every three weeks. The patient completed six cycles of VAC/IE chemotherapy.

Restaging imaging subsequently demonstrated a new hepatic metastatic lesion measuring 2.0 × 1.8 cm. The case was re-discussed at the multidisciplinary team meeting, and the chemotherapy regimen was changed to vincristine, irinotecan, and temozolomide (VIT). Vincristine (1.5 mg/m²), irinotecan (20 mg/m²), and temozolomide (100 mg/m²) were administered on days 1-5. The patient subsequently received eight cycles of VIT chemotherapy.

After the completion of six cycles of VAC/IE followed by eight cycles of VIT chemotherapy (14 cycles in total), repeat imaging demonstrated a 73.9% reduction in the primary tumour size, with the residual tumour measuring 5.7 × 5.8 × 5.3 cm as shown in Figure 1B. The IVC thrombus reduced to 1.7 cm. The liver lesion decreased to a size of 1.7 × 1.2 cm.

Surgical management

Following reassessment, the patient underwent left radical nephrectomy with IVC thrombectomy and segment IV liver resection with cholecystectomy as shown in Figure 1C. The planes were better delineated intraoperatively.

Postoperative recovery was uneventful. Drains were removed on postoperative day 4. She was discharged on postoperative day 6. In the biopsy-first approach, neoadjuvant chemotherapy resulted in significant tumour downsizing (73.9%) and reduction in IVC thrombus burden, facilitating complete surgical resection with minimal perioperative morbidity. Histopathology revealed left renal Ewing's sarcoma with 90% tumour necrosis with resection margin free of tumour. Cells were uniform small round with finely stippled chromatin, inconspicuous nucleoli, and scant cytoplasm with a few having nuclear clearing. Brisk mitosis was seen. On IHC, the tumour cells were positive for CD99 and negative for CD45 as shown in Figure 2.

**Figure 2:**
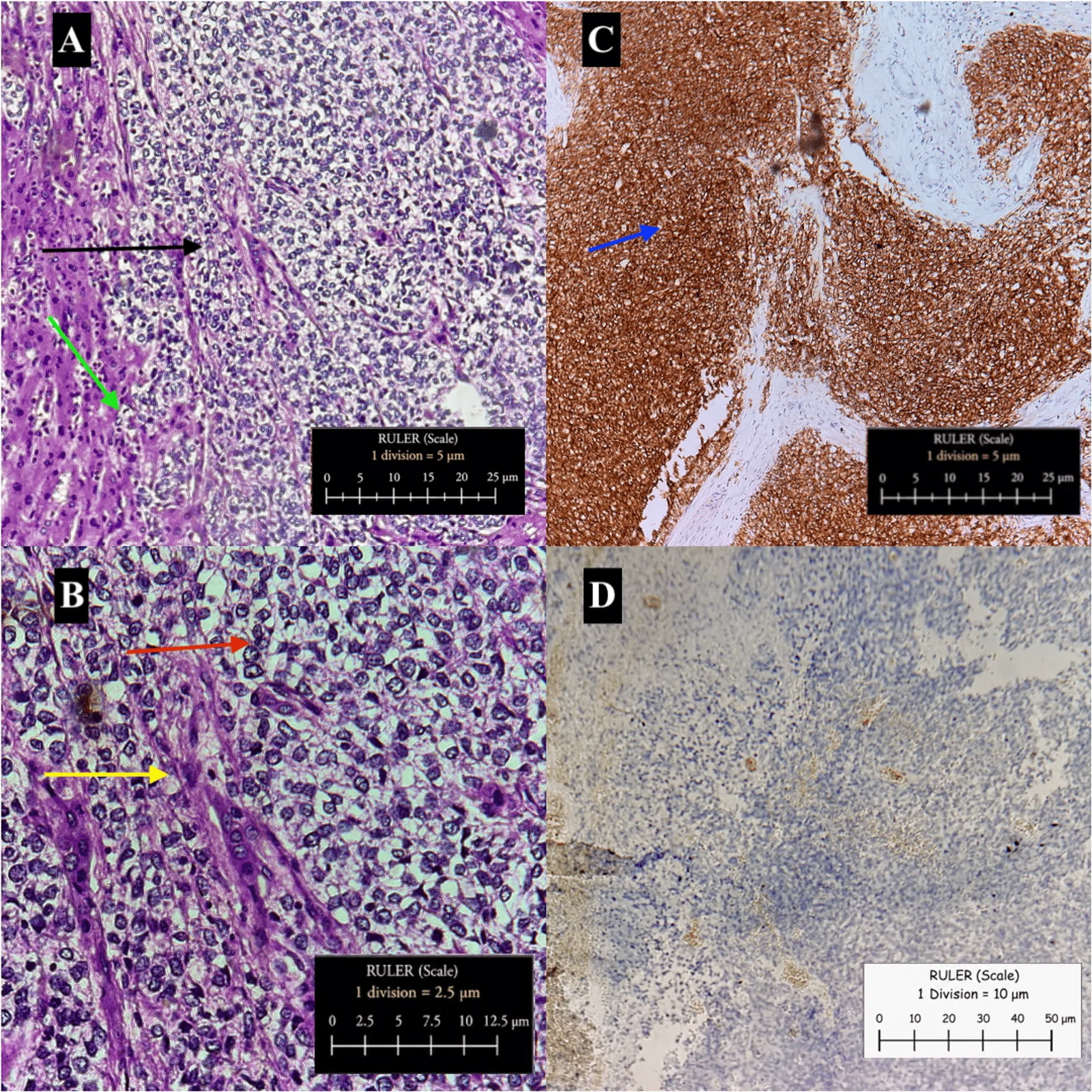
Histopathological images of Case 1 (A) Black arrow showing nests of small round blue cell tumour from the renal mass tissue and green arrow showing fibroblastic atroma (H&E stain, 20× magnification). (B) Red arrow showing tumour cells with a high nuclear-to-cytoplasmic ratio from the renal mass tissue and yellow arrow showing fibrous septa (H&E stain, 40× magnification). (C) Blue arrow showing diffuse positivity for CD99 (CD99 stain (IHC), 20× magnification). (D) FL1 was non-diagnostic (FL1 stain, 10× magnification). H&E: hematoxylin and eosin; IHC: immunohistochemistry

Following medical oncology consultation, she was started on VIT. She is asymptomatic and continuing chemotherapy. At the one-year follow-up, she remains disease-free.

Case 2

A 29-year-old woman presented with left-sided abdominal pain and gross painless haematuria without clots for 15 days. Table 2 shows the baseline investigations of the second patient with decreased haemoglobin and borderline raised serum creatinine.

**Table 2:** Baseline investigations of the second patient Baseline investigation showing anaemia and raised creatinine.

Parameter	Obtained value	Reference value
Haemoglobin	7.1 g/dL	11-15 g/dL
Serum creatinine	1.20 mg/dL	0.45-1 mg/dL

Coronal section contrast-enhanced CT of the abdomen and thorax of this patient revealed a 9.5 × 10.8 × 15.5 cm mass with level II IVC thrombus. No distant metastases were detected as shown in Figure 3A.

**Figure 3:**
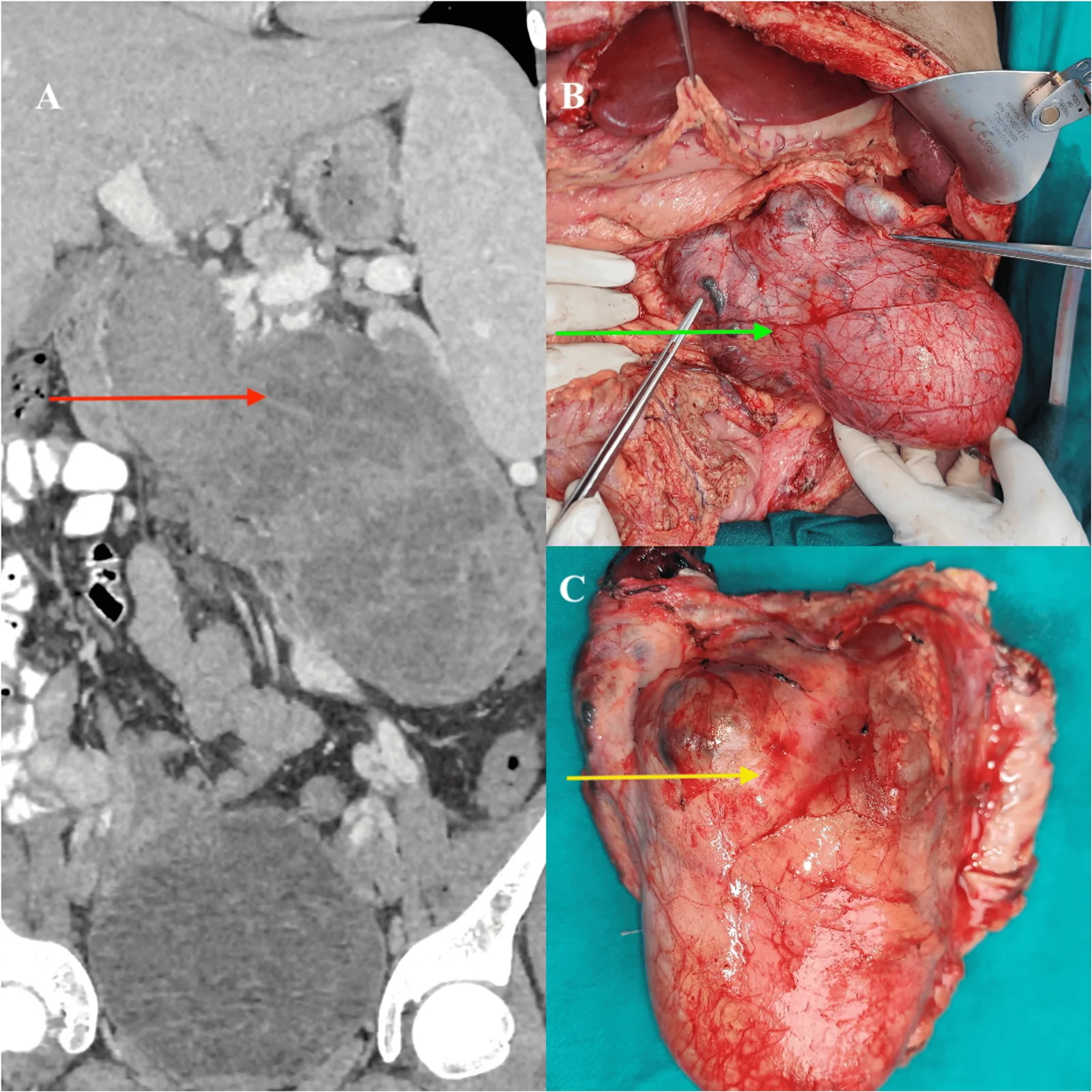
CT scan and specimen images of Case 2 (A) CT scan coronal images with the red arrow showing the left renal mass with IVC thrombus. (B) Specimen image with the green arrow showing the intraoperative intact specimen. (C) Specimen image with the yellow arrow showing the postoperative specimen of the kidney with mass. IVC: inferior vena cava

Management strategy

She had ongoing haematuria and a fall in haemoglobin to 6.0 g/dL. She was discussed in a multidisciplinary discussion. Giving importance to the falling haemoglobin and significant thrombus burden, a plan was made for blood transfusion followed by urgent surgical intervention.

Surgical intervention

The patient underwent open left radical nephrectomy with IVC thrombectomy. The thrombus extended 4 cm below the hepatic vein confluence within the intrahepatic IVC. Following liver mobilisation, the thrombus was successfully removed en bloc. Figure 3B shows the intact left renal mass. The postoperative specimen of the renal mass is shown with the thrombus (Figure 3C).

The patient developed a Clavien-Dindo grade IIIb postoperative complication in the form of persistent high-output chylous leak (4 L/day), confirmed by elevated drain fluid triglycerides. Initial management included interventional radiology-guided lymphangiography and glue embolisation which she underwent twice.

However, persistent leak required exploratory laparotomy with retroperitoneal lymphatic trunk ligation. During surgery, an iatrogenic ileal perforation due to adhesions was repaired. Subsequently, the patient developed burst abdomen, which was then managed by mesh laparostomy and en masse closure with tension wiring. She was eventually discharged on postoperative day 63.

Histopathology

Final histopathology confirmed Ewing's sarcoma of the kidney (pT1) with strong CD99 positivity and patchy NKX2.2 expression. Tumour invasion into the renal sinus, pelvis, and renal vein was noted. Histopathological images are shown in Figure 4.

**Figure 4:**
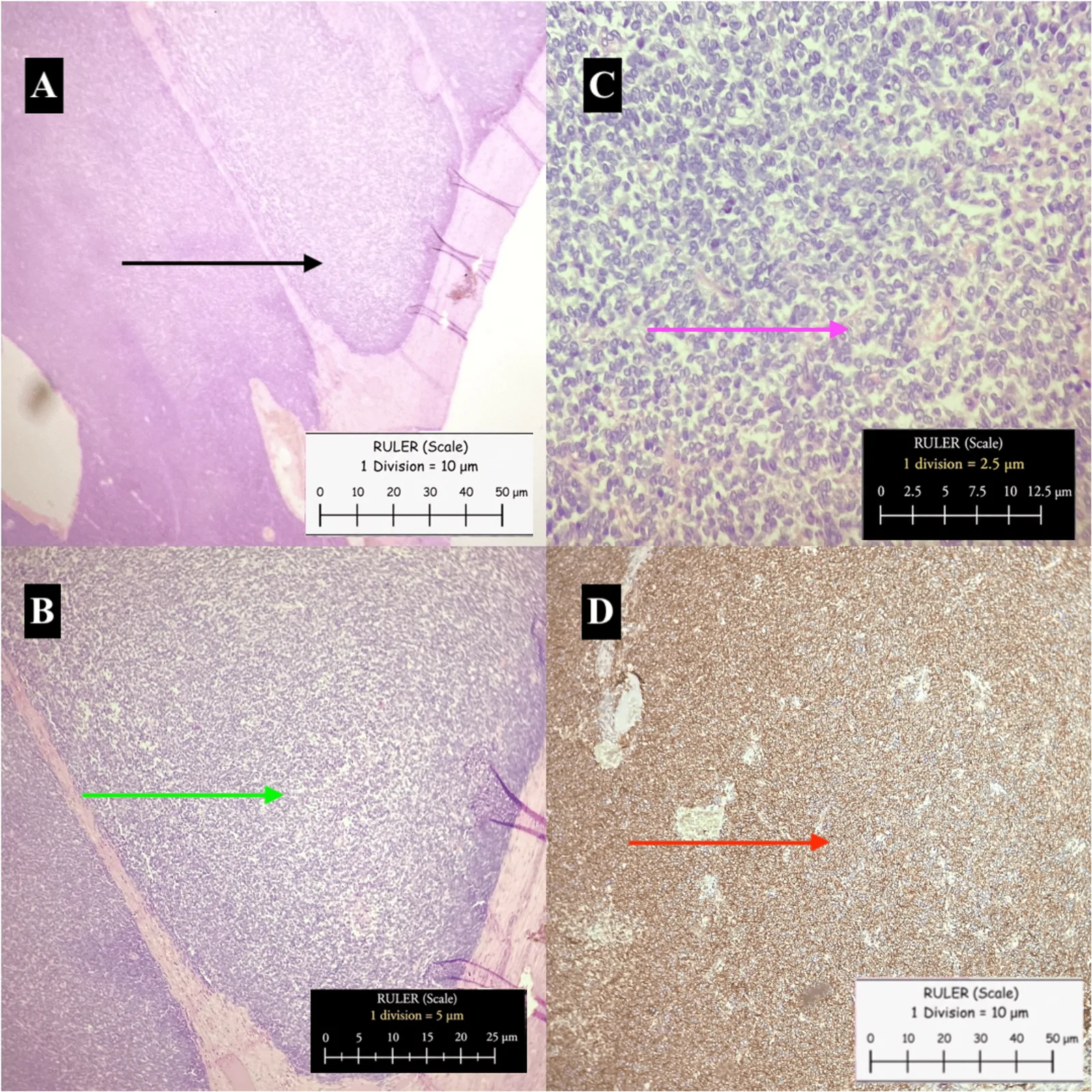
Histopathological microscopic images of Case 2 (A) Black arrow showing sheets of malignant tumour arranged in sheets (H&E stain, 10× magnification). (B) Green arrow showing sheets and nests of malignant small blue tumour cells arranged in sheets (H&E stain, 20× magnification). (C) Pink arrow showing tumour cells with scant cytoplasm and high N:C ratio (H&E stain, 40× magnification). (D) Red arrow showing diffuse CD99 positivity (CD99 stain, 10× magnification). N:C: nuclear-to-cytoplasmic; H&E: hematoxylin and eosin

Adjuvant chemotherapy

Adjuvant VAC/IE chemotherapy was initiated after recovery. However, chemotherapy initiation was delayed due to wound healing complications. At the one-year follow-up, imaging showed no evidence of recurrence or metastasis, and the patient demonstrated gradual functional recovery.

## Discussion

Primary renal Ewing's sarcoma is an exceptionally uncommon malignancy belonging to the Ewing sarcoma family of tumours (ESFT). It accounts for fewer than 1% of primary renal neoplasms and predominantly affects adolescents and young adults, with a median age at diagnosis of approximately 28-30 years [3,4]. Owing to its rarity and aggressive biological behaviour, most available evidence is derived from isolated case reports and small case series rather than prospective studies. Patients frequently present with abdominal or flank pain, haematuria, a palpable abdominal mass, or constitutional symptoms, while a substantial proportion have metastatic disease at presentation, reflecting the tumour's high metastatic potential [5].

Radiologically, renal Ewing's sarcoma lacks pathognomonic imaging characteristics. Contrast-enhanced CT usually demonstrates a large heterogeneous renal mass with areas of necrosis, haemorrhage, and irregular enhancement, findings that closely resemble advanced RCC [6]. Extension into the renal vein and IVC, although well recognised in RCC, has also been described in renal Ewing's sarcoma, making preoperative differentiation particularly challenging. Consequently, many patients undergo radical nephrectomy with a presumed diagnosis of RCC, and the diagnosis of Ewing's sarcoma is established only after histopathological examination. In both of our patients, the presence of a large renal mass with level II IVC thrombus initially raised the suspicion of RCC, illustrating this important diagnostic dilemma [7,8].

Histopathologically, renal Ewing's sarcoma is characterised by sheets of uniform small round blue cells with scant cytoplasm and frequent areas of necrosis. Immunohistochemically, diffuse membranous CD99 positivity remains the hallmark finding, while NKX2.2 has emerged as a useful complementary marker with relatively high sensitivity and specificity [9]. Nevertheless, neither marker is entirely specific, and molecular confirmation by demonstrating an EWSR1 gene rearrangement, most commonly the EWSR1-FLI1 fusion transcript, remains the diagnostic gold standard whenever available [10]. Although molecular testing could not be performed in our patients because of resource limitations, the characteristic morphology together with diffuse CD99 positivity and NKX2.2 expression strongly supported the diagnosis.

The management of renal Ewing's sarcoma differs fundamentally from that of RCC because it is considered a systemic disease even when apparently localised. Current treatment strategies are extrapolated from protocols developed for skeletal and extraosseous Ewing's sarcoma and advocate multimodal therapy consisting of intensive multi-agent chemotherapy combined with complete surgical excision, with or without radiotherapy depending on surgical margins and local disease control [11-13]. Contemporary chemotherapy regimens employing alternating vincristine, doxorubicin, and cyclophosphamide with ifosfamide and etoposide (VDC/IE or VAC/IE) have significantly improved survival in localised Ewing's sarcoma through the eradication of occult micrometastatic disease and improved local control. The timing of chemotherapy relative to surgery, however, remains uncertain in primary renal Ewing's sarcoma because of the limited number of reported cases. In conventional RCC, radical nephrectomy with IVC thrombectomy remains the standard treatment for resectable disease. Conversely, in Ewing's sarcoma, neoadjuvant chemotherapy is routinely administered because of its high chemosensitivity and the need for early systemic treatment. Several cooperative studies in osseous Ewing's sarcoma have demonstrated that preoperative chemotherapy improves resectability and facilitates limb- or organ-preserving surgery and that the percentage of tumour necrosis after chemotherapy is an important prognostic indicator. Histological necrosis exceeding 90% is consistently associated with improved event-free and overall survival.

The contrasting treatment sequences in our two patients highlight the importance of individualising the timing of systemic therapy and surgery in renal Ewing's sarcoma with IVC thrombus. In the first patient, the absence of distant metastases at presentation, preserved performance status, and renal function, together with extensive local disease and a radiologically atypical renal mass with significant vascular involvement, prompted the consideration of a diagnosis other than conventional RCC. Given that the histological diagnosis could substantially alter the initial treatment strategy, image-guided biopsy was performed before surgery. Histopathological confirmation of Ewing's sarcoma allowed the multidisciplinary planning and initiation of systemic chemotherapy. The subsequent reduction in the primary tumour and IVC thrombus resulted in better-defined tissue planes at surgery and facilitated definitive resection.

In contrast, the second patient initially had a radiological diagnosis suggestive of RCC but developed persistent haematuria with a progressive fall in haemoglobin despite transfusion, in the setting of a substantial IVC thrombus burden. After multidisciplinary discussion, urgent upfront nephrectomy and IVC thrombectomy were considered necessary because of the ongoing clinical deterioration. Although complete resection was achieved, the postoperative course was complicated by a persistent high-output chylous leak requiring interventional and subsequent surgical management, followed by additional wound-related morbidity. These complications substantially delayed recovery and consequently postponed the initiation of systemic chemotherapy. Thus, while both patients ultimately underwent complete surgical management, the biopsy-first approach in the clinically stable patient permitted systemic therapy before surgery and tumour/thrombus downsizing, whereas the surgery-first approach in the clinically deteriorating patient prioritised immediate control of the symptomatic disease but was followed by significant postoperative morbidity and delayed systemic treatment. These observations do not establish the superiority of one sequence over the other, but illustrate how clinical stability, symptom burden, tumour extent, and the anticipated impact of systemic therapy should be considered when determining the optimal treatment sequence.

More recent case reports and institutional series have suggested that image-guided biopsy may be considered in young patients with atypical renal masses, particularly when the histological diagnosis is likely to alter the initial treatment strategy. More broadly, rare renal and perirenal entities may present with indeterminate imaging features and mimic neoplastic lesions, underscoring the limitations of imaging alone and the importance of clinicoradiological-pathological correlation. In our cases, establishing the diagnosis before surgery facilitated multidisciplinary treatment planning and enabled systemic chemotherapy to be initiated before definitive surgery in the clinically stable patient [14].

These two cases should not be interpreted as evidence that neoadjuvant chemotherapy is universally superior to immediate surgery. Rather, they highlight the importance of individualised treatment sequencing based on tumour biology, symptom burden, haemodynamic stability, thrombus characteristics, and multidisciplinary assessment. Patients with life-threatening haemorrhage, impending thromboembolic complications, or rapidly deteriorating clinical status may require urgent surgical intervention despite the potential benefits of neoadjuvant therapy. Conversely, clinically stable patients with atypical radiological features should be considered for preoperative biopsy because confirmation of Ewing's sarcoma fundamentally changes the therapeutic strategy. The principal limitation of this report is its small sample size, which precludes definitive conclusions regarding the superiority of either management pathway. Nevertheless, because renal Ewing's sarcoma with IVC thrombus is exceptionally rare, comparative descriptions of different treatment sequences remain valuable. Future multicentre collaborative registries are needed to define optimal patient selection for neoadjuvant chemotherapy, establish standardised treatment algorithms, and determine the long-term oncological impact of treatment sequencing.

Given the rarity of renal Ewing's sarcoma and the limited number of reported cases with IVC tumour thrombus, several questions remain unresolved. Prospective or multicentre studies are needed to identify patients most likely to benefit from neoadjuvant chemotherapy, define the clinical and radiological factors that should favour biopsy-first management, establish reproducible treatment pathways, and determine whether treatment sequencing influences long-term oncological outcomes. The proposed workflow in Figure 5 is therefore intended as a pragmatic, hypothesis-generating framework derived from the clinical scenarios described in this report and the currently available literature, rather than as a definitive treatment guideline.

**Figure 5:**
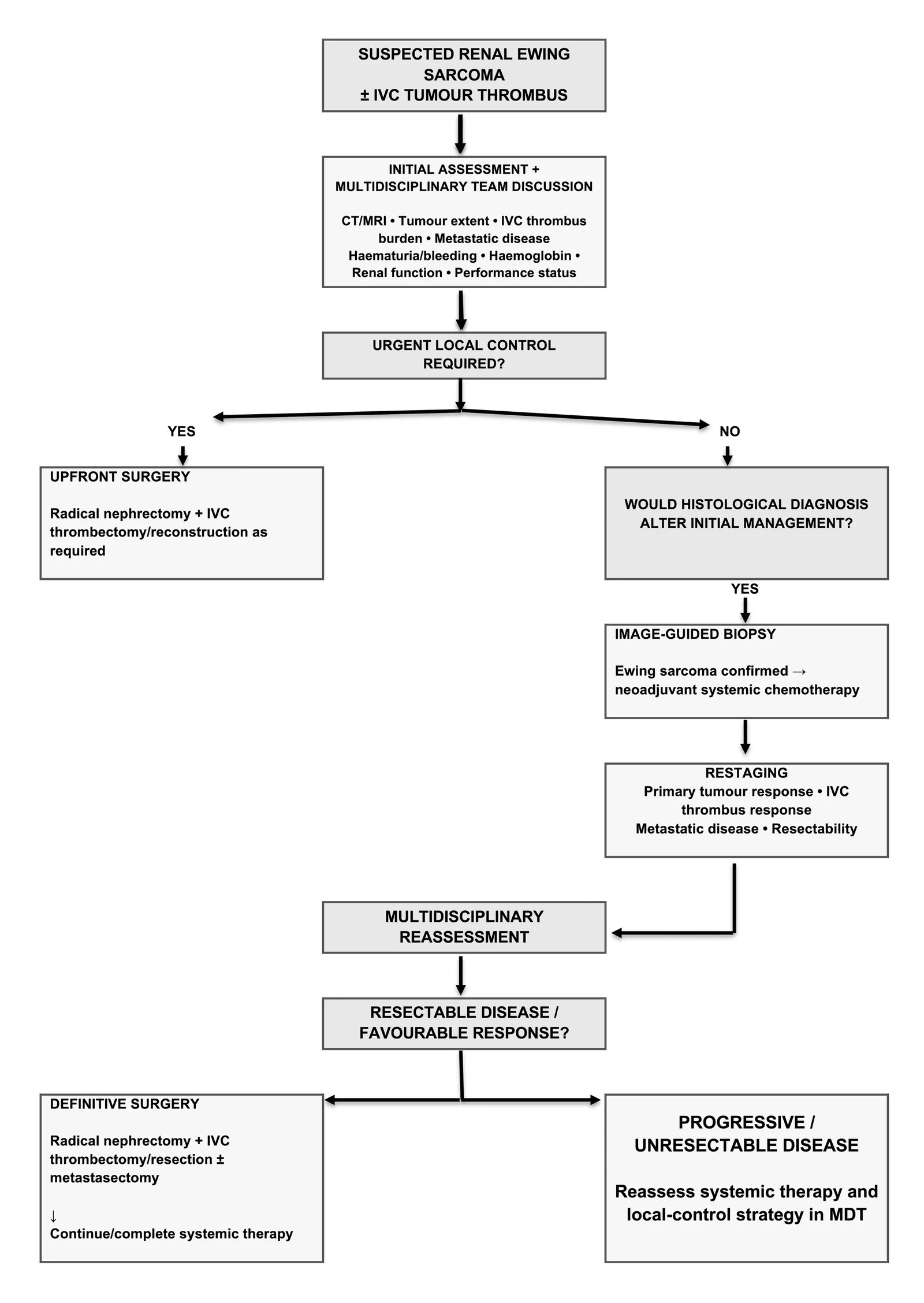
Proposed diagnostic and treatment workflow for renal Ewing's sarcoma with IVC tumour thrombus The algorithm illustrates a pragmatic approach to treatment sequencing based on clinical stability, symptom burden, tumour extent, IVC thrombus burden, and the potential impact of histological diagnosis on initial management. The workflow is hypothesis-generating and is not intended to represent a validated treatment guideline. IVC: inferior vena cava; MDT: multidisciplinary team

## Conclusions

This case series highlights the potential advantages of a biopsy-first approach in young patients with suspected renal Ewing's sarcoma and IVC thrombus. Early histological diagnosis enabled neoadjuvant chemotherapy, leading to marked tumour cytoreduction, thrombus regression, and favourable surgical outcomes in one patient, whereas upfront surgery in the second patient was associated with substantial postoperative morbidity. Although larger studies are needed, these cases suggest that preoperative tissue diagnosis and multidisciplinary planning may play a pivotal role in optimizing outcomes for this rare and aggressive malignancy.
